# Review of the Real-Time Monitoring Technologies for Lithium Dendrites in Lithium-Ion Batteries

**DOI:** 10.3390/molecules29092118

**Published:** 2024-05-03

**Authors:** Yifang Liang, Daiheng Song, Wenju Wu, Yanchao Yu, Jun You, Yuanpeng Liu

**Affiliations:** 1 Key Laboratory of Green Chemical Engineering and Technology of College of Heilongjiang Province, College of Materials Science and Chemical Engineering, Harbin University of Science and Technology, Harbin 150080, Chinayoujunjun@126.com (J.Y.); 2 National Key Laboratory of Science and Technology on Advanced Composites in Special Environments, Center for Composite Materials and Structures, Harbin Institute of Technology, Harbin 150080, China

**Keywords:** lithium dendrite, lithium-ion battery, real-time monitoring technology, in situ/operando characterization, sensor

## Abstract

Lithium-ion batteries (LIBs) have the advantage of high energy density, which has attracted the wide attention of researchers. Nevertheless, the growth of lithium dendrites on the anode surface causes short life and poor safety, which limits their application. Therefore, it is necessary to deeply understand the growth mechanism of lithium dendrites. Here, the growth mechanism of lithium dendrites is briefly summarized, and the real-time monitoring technologies of lithium dendrite growth in recent years are reviewed. The real-time monitoring technologies summarized here include in situ X-ray, in situ Raman, in situ resonance, in situ microscopy, in situ neutrons, and sensors, and their representative studies are summarized. This paper is expected to provide some guidance for the research of lithium dendrites, so as to promote the development of LIBs.

## 1. Introduction

Lithium-ion batteries (LIBs) possess the merit of high energy density and have been commonly applied in various energy storage devices [1,2,3,4,5], whereas their life and safety performance cannot meet demands, which is not conducive to their further development [6,7]. The growth of lithium dendrites on the anode surface is the cause of short life and poor safety [8,9,10]. During the operation of the battery, the lithium ions in the electrolyte will obtain electrons from the external circuit and deposit them on the anode in the form of dendrites. The surface of the original lithium is uneven, and these uneven surface states lead to uneven charge distribution [11]. The uneven deposition of lithium ions will increase with the cycle, and finally the formation of lithium dendrites on the anode surface will occur. As a result of the concentration of ion flux, lithium is preferentially deposited at the tip during the deposition process, resulting in increased roughness of the anode surface, which leads to the formation of more dendrites [12]. Lithium dendrites will grow further in the process of charge and discharge cycling, and the internal short-circuit will be caused when the dendrites penetrate the separator or solid-state electrolytes, which may lead to fire or even explosions [13,14]. In addition, if the thin neck of the dendrites breaks during the cycle, the newly exposed lithium surface will immediately react with the electrolytes to form a solid electrolyte interphase (SEI) with poor electronic conductivity, which will lose the conductive connection with the anode and cannot continue the reaction, and is named dead lithium [15,16]. Dead lithium will produce irreversible consumption of lithium and electrolytes, resulting in poor Coulombic efficiency (CE) and irreversible capacity loss of the battery [17,18].

Recently, several methods have been studied in depth to solve the problem of uneven formation of lithium dendrites, including the development of artificial SEI film, the study of a three-dimensional current collector, the design of liquid electrolyte additives, and the use of solid-state electrolytes [19], whereas we must first study the reaction mechanism to solve this issue fundamentally. Therefore, in order to obtain LIBs with higher safety in practical applications, it is requisite to carry out research on the growth of lithium dendrites in depth. In recent years, numerous usual characterization approaches have been utilized to study lithium dendrites by investigating the structural evolution of electrode materials during the process of charge and discharge [20]. Ex situ/traditional characterization methods usually require disassembly of the battery to obtain a sample, and can only be used to study the composition, variation in material structure, and morphology before and after the cycling process, but cannot be used to monitor and observe the actual phenomenon in real time during the cycle, and also cannot be employed to investigate the reaction mechanism during electrochemical reactions [21]. Hence, the exploitation of real-time monitoring technology is vital for the study of LIBs. Real-time monitoring technology can observe the variations of the composition, structure, and morphology of electrode materials with time during cycling, and promote the study of the reaction mechanism of electrode materials. Furthermore, real-time monitoring technology can elucidate the phenomena of lithium dendrite growth, dead lithium generation, and SEI interface evolution. 

**Figure 1:**
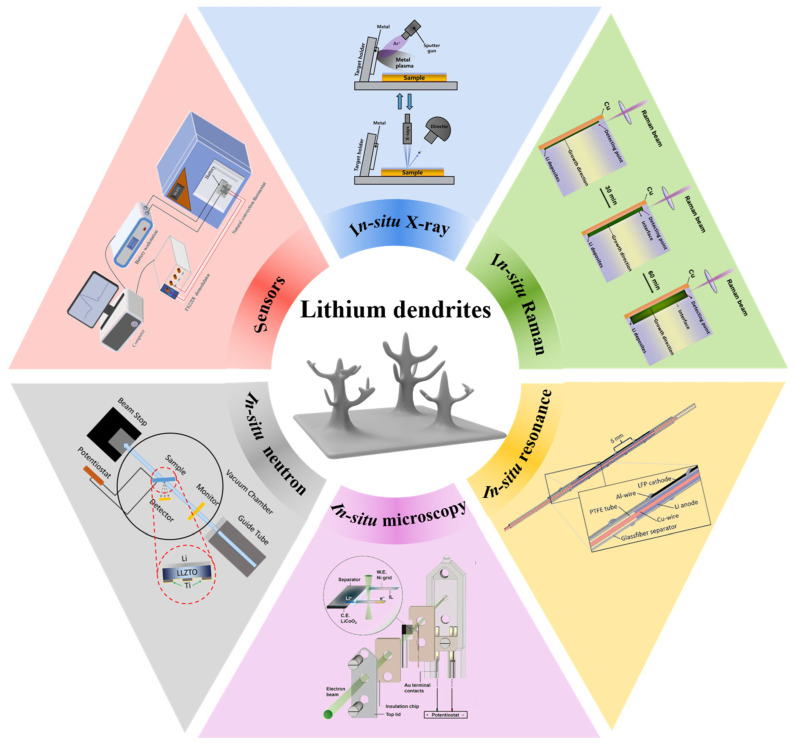
Schematic diagram of real-time monitoring technology for lithium dendrites in LIBs. Reproduced with permission [22]. Copyright 2023, Wiley. Reproduced with permission [23]. Copyright 2019, Nature Portfolio. Reproduced with permission [24]. Copyright 2015, Royal Society of Chemistry. Reproduced with permission [25]. Copyright 2021, Wiley. Reproduced with permission [26]. Copyright 2019, Elsevier. Reproduced with permission [27]. Copyright 2022, Elsevier Science SA.

Herein, the application of real-time monitoring technology in LIBs is reviewed. We first outline the growth mechanism of lithium dendrites, and then discuss in detail the application of real-time monitoring technology in LIBs, including in situ/operando characterization technologies and sensors, as shown in Figure 1. We also overview the main uses, merits, and drawbacks of real-time monitoring technologies in Table 1. The growth mechanism of lithium dendrites obtained from various advanced real-time monitoring technologies is of extraordinary value for achieving safe, high-performance, and long-life LIBs. Finally, we further present the future exploitation orientation of real-time monitoring technology. This review is anticipated to guide the investigation of lithium dendrites in the future.

## 2. Mechanism of Lithium Dendrite Growth

The dendrites are deposited in the shape of three-dimensional mosses, shrubs, trees, and even needles during the cycling [45]. Studies of the mechanism of lithium dendrite growth can be traced back to the early 1960s. Barton and Bockris presented the comprehensive model of dendrite growth in 1962 [46]. The theory is that the plating on the electrode protrusions is faster because spherical diffusion conditions predominate over linear diffusion conditions. The dendrite growth rate is the highest relative to the tip radius. The model assumes that the electrochemical reaction kinetics are linear and the dendrites are static hemispheres. Inertial and mechanical forces are ignored. Diggle et al. modified this theory by allowing for higher overpotentials and relaxing the assumption of static dendrite [47]. Aogaki and Makino presented that the unstable ion concentration on the electrode surface leads to the surface bulge, which in turn intensifies the instability of electrolyte concentration, in 1981 [48]. In 1987, Bruce and Vincent discovered that the overpotential between the two electrodes depended on the initial potential and the ion concentration [49].

Chazalviel presented that the nucleation and growth of lithium dendrites can be illustrated by ion diffusion and migration behavior [50]. The theory suggested that the depletion of ions near the electrode surface destroys the charge balance on the electrode surface and leads to the existence of space charge, which is the cause of dendrites. Nevertheless, convection was ignored in this theory. Fleury et al. revised this theory in 1992 by adding the presence of electric convection during electrodeposition, where the deposition is regarded as equidistant lines and infinitely thin needles growing between two plates [51]. Brissot et al. proposed in 1999 that the growth of lithium dendrites is related to the current density [52]. In situ observation of lithium dendrite deposition was conducted under constant current conditions, while the change of battery potential, the evolution of dendrites, and the change of ion concentration in the electrolytes surrounding the dendrites were measured. Two different patterns of dendrite growth were observed at different current densities. Dendrites begin to grow when the ion concentration of the anode drops to zero at high current densities, while local non-uniformity appears to play a crucial role at low current densities. Monroe and Newman modeled the dendrite growth and included the influence of dendrite tip curvature in the dendrite growth kinetics [53]. Studies have shown that dendritic growth always slows as the current density decreases. 

In recent years, many theories of lithium dendrite growth have been proposed. Wang et al. presented a surface energy model to investigate lithium dendrite growth [54]. The surface energy of the substrate consists of the surface energy of the deposited film and the electrical potential energy. When the surface energy of the substrate is lower than that of the deposited film, lithium dendrites will be formed during electrodeposition. Sun et al. acquired visual evidence of the dynamic process of Li dendrite formation and growth by in situ microscopic observation of Li deposition in solid-state electrolytes [55]. It can be seen that Li nucleates and grows directly within a solid-state electrolyte, causing its structure to crack. This phenomenon should be induced due to the existence of P- and S- based crystal defects in Li_3_PS_4_ solid-state electrolytes, which is in agreement with the theoretical calculation and observation of cryo-transmission electron microscopy. This view provides significant perceptions of the growth mechanism of lithium dendrites. Wang et al. indicated that local lithium-ion flux and deposition active site can determine the formation of lithium dendrite, which are related to the content and type of defects in solid-state electrolytes [56]. Furthermore, the defect areas show more quick lithium deposition kinetics and greater nucleation tendency.

The basic mechanism of lithium dendrite growth consists of the following three stages [57]. In the first stage, an SEI film is formed by the reaction. An even SEI can passivate the anode and prevent further reactions, but SEIs that spontaneously form are usually non-uniform. In the second stage, the nucleation stage, the non-uniform deposition continues to accumulate, and the uneven SEI provides nucleation sites for dendrite formation, appearing in whisker protrusion, until destroying the SEI film. Finally, the negative charge will aggregate on the tip and lithium ions will be preferentially reduced on the tip, thus forming dendrites in the deposition process. Fresh lithium is exposed to the electrolytes each cycle, eventually depleting the electrolytes. The dendrites sustain to grow and ultimately puncture the separator or solid-state electrolytes after iterative cycles, leading to battery failure. 

The growth of lithium dendrites causes various performance issues in the battery, such as capacity degradation, temperature rise, short circuits, and so on [15,17]. Therefore, it is vital to observe the electrode material variations, SEI interface evolution, and lithium dendrite growth with time during cycling through real-time monitoring technology in order to analyze the above issues. In terms of the capacity degradation of the battery, it is closely related to the structural and morphology changes of SEI and electrode materials, which could be detected through in situ XRD [58], in situ XPS [22], in situ Raman [30], in situ NMR [32], in situ NDP [59], operando SANS [41], in situ SEM [36], in situ TEM [60], and so on. In terms of temperature rise, it could be directly detected by sensors [42]. Moreover, in terms of short circuits, it is closely related to the microstructure of the lithium growth during lithium deposition and the evolution of the sedimentary phase of lithium metal, which could be detected by in situ OM [61], in situ SEM [36], in situ TEM [60], in situ NI [62], in situ AFM [63], and so on. More detailed discussions of real-time monitoring technologies will be presented in the next section.

## 3. Real-Time Monitoring Method of Lithium Dendrites

### 3.1. In Situ/Operando Characterization

Numerous characterization techniques are employed to investigate the structure and morphology of lithium dendrites. However, the ex situ characterization can only be used for electrode testing before and after the reaction, which limits the study of the detailed structural variations of lithium dendrites and the stages of the reaction process [35]. Besides, the results of ex situ characterization may not completely reveal what is really happening during electrochemical processes because of the sensitivity of the electrode to the humidity and air in the environment. Thus, in order to obtain the growth process of lithium dendrites under real operating conditions, researchers have employed in situ characterization to monitor the growth of lithium dendrites in real time [64,65]. In situ characterization can allow real-time observation of the growth process of lithium dendrites without disassembling the test battery [66]. The comparison of each in situ characterization technique with the corresponding ex situ characterization technique is shown in Table 2.

#### 3.1.1. In Situ/Operando X-ray Spectroscopy

X-ray characterization technology is applied to the investigation of the reaction mechanism of electrode materials because it can provide the structure and phase transition information [66]. In situ/operando X-ray characterization technology can monitor electrode materials in real time during the process of battery charging and discharging and has been widely applied to the investigation of lithium metal batteries in recent years. Widely used in situ/operando X-ray characterization technologies mainly include in situ X-ray diffraction (XRD) and in situ/operando X-ray photoelectron spectroscopy (XPS).

(1) In situ XRD spectroscopy

X-ray features the particular sensitivity of Bragg diffraction and strong penetration. XRD is an analytical method that uses X-ray diffraction formed by crystals to acquire information such as the morphology or structure of atoms or molecules inside materials and the composition of materials, and is broadly used in the study of the material phase and lattice due to its advantages of no destruction to the sample, high detection speed, high precision, and no contamination [28]. For lithium metal batteries, XRD is frequently utilized to investigate the phase transitions, crystallinity, and crystal structure of solid electrolytes and electrodes. In situ XRD technology means the XRD test during the process of battery charging and discharging, monitoring the structural changes of solid electrolyte and electrode materials in real time during the cycle process, and investigating the reaction mechanism during the cycle process [58].

**Figure 2:**
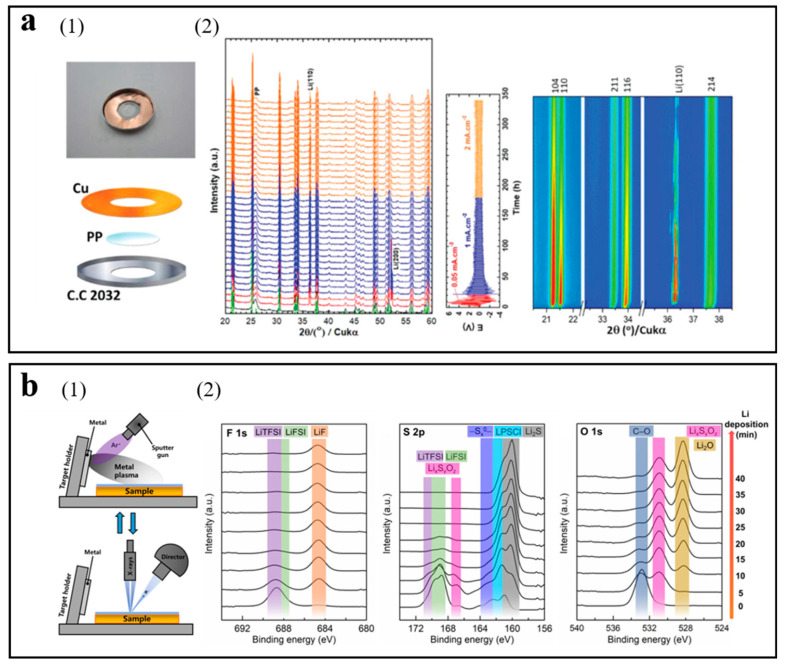
(**a**) Battery configuration and spectra of in situ XRD. (1) The battery configuration of the in situ XRD test. (2) In situ XRD spectra, the corresponding change of voltage with time, and contour plot of expanding 2θ ranges exhibiting the texturing of Li(110) and up-shift of 2θ. Intensity: blue = minimum, red = maximum. Reproduced with permission [67]. Copyright 2020, Wiley. (**b**) Experimental setup and spectra of in situ XPS. (1) Schematic diagram of in situ XPS with lithium deposition. (2) In situ XPS spectra with time of lithium deposition. Reproduced with permission [22]. Copyright 2023, Wiley.

Paolella et al. presented a preparation method of a self-standing and ultrathin (≈70 μm) NASICON-type Li_1_._5_Al_0_._5_Ge_1_._5_(PO_4_)_3_ (LAGP) electrolyte and collected the in situ XRD spectra of lithium symmetrical batteries during the cycle process [67]. The battery configuration of the in situ XRD test is shown in Figure 2(a1). As shown in Figure 2(a2), the preferential orientation growth of the lithium metal surface along the (110) crystal plane was observed from the in situ XRD spectra. The (110) plane with more steps and low-coordinated surface atomic kinks can afford preferred sites for Li metal deposition/dissolution. Lu et al. proposed that Ag nanoparticles were encapsulated inside nitrogen-doped carbon hollow spheres to construct a three-dimensional conductive host with hierarchical lithiophilicity, which is certified to facilitate even Li deposition by in situ XRD spectra [68].

(2) In situ/operando XPS spectroscopy

XPS is a technology for investigating the surface, microstructure, and deep distribution of materials. Using photons in the X-ray range to induce the emission of core electrons, XPS enables the provision of information about the elemental composition, molecular structure, chemical state, and atomic valence bonds of a compound through measuring the electron binding energy [29]. For lithium metal batteries, XPS is ideal for evaluating the chemical structure of the electrode/electrolyte interface. In situ/operando XPS can directly investigate the chemical changes of the interface under electrochemical conditions, as well as analyze conducive information such as chemical composition and surface/interface structure.

Huo et al. reasonably designed the solid electrolyte interface of thin polymer/thiophosphate to further dendrite-free lithium metal batteries [22]. In situ XPS was utilized to study the SEI components, and the experimental device was exhibited in Figure 2(b1). LiF-rich SEI was demonstrated by in situ XPS in Figure 2(b2), which reveals uniform lithium deposition. Wood et al. exploited operando XPS to monitor changes in the surface of lithium metal and investigate the formation and evolution of the Li/Li_2_S-P_2_S_5_ solid-electrolyte interphase during the cycling process as well as the chemical state and compositional information [69]. The operando XPS results revealed that Li_3_PO_4_ inhibits Li^+^ migration, which was not conducive to the uniform deposition of lithium.

#### 3.1.2. In Situ Raman Spectroscopy

The Raman effect arises from the inelastic scattering of monochromatic probe light when it interacts with the material [70]. The crystallinity, chemical structure, phase, and molecular interactions of the sample can be analyzed by Raman spectroscopy. In situ Raman spectroscopy is a non-destructive technology that can be employed to analyze amorphous or weakened crystallized compounds compared to in situ XRD, as well as investigate the mechanical, structural, and chemical changes of electrodes, electrolytes, and electrode/electrolyte interfaces during the battery charging and discharging process [30]. Nevertheless, in situ Raman cannot be used to explore Li metal because the Raman shift is sensitive to non-polar bonds and only depends on the energy level structure of the intrinsic vibration and rotation of the molecule [31]. Fortunately, exploring changes in the interface between the electrolyte and the lithium metal can probe the deposition mechanism. 

**Figure 3:**
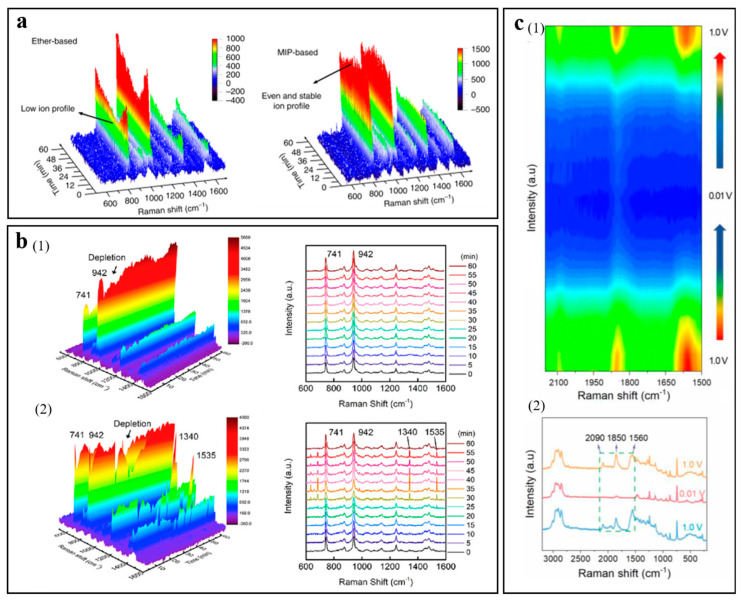
(**a**) In situ Raman spectra of ether-based and introduced montmorillonite electrolytes. The TFSI^−^ band corresponds to the Raman band at 720–760 cm^−1^. Reproduced with permission [23]. Copyright 2019, Nature Portfolio. (**b**) In situ Raman spectra of the electrolyte without (1) and with (2) graphene quantum dots. Reproduced with permission [71]. Copyright 2020, Elsevier. (**c**) In situ Raman spectra of the battery with electrode based on a scaffold (polyethyleneimine @Ag@Cu). (1) Contour map. (2) Raman spectra. Reproduced with permission [74]. Copyright 2023, Elsevier.

Chen et al. discovered that adding the montmorillonite into the ether-based electrolyte can optimize the distribution of Li^+^ in the electrolyte as a result of the ionic self-concentrate on the montmorillonite, which can achieve homogeneous deposition of Li, resulting in dendrite-free Li deposition [23]. As shown in Figure 3a, the signal strength change of TFSI^−^ is negligible by in situ Raman monitoring on the electrochemical interface, illustrating uniform distribution of Li^+^, and generating a homogeneous lithium deposition. Hu et al. proposed a novel tactic to inhibit dendrite growth by adding graphene quantum dots into the electrolyte [71]. The in situ Raman spectroscopy exhibits the enrichment of the graphene quantum dots at the interface between electrode and electrolyte in Figure 3b, resulting in the dendrite-free Li deposition. Liu et al. designed a three-dimensional honeycomb-like hierarchical nitrogen-doped framework as a substrate for lithium deposition, where Li^+^ can be uniformly distributed due to the adsorption of N-containing functional groups [72]. In situ Raman was utilized to investigate the Li stripping/plating behaviors, which revealed the disappearance of numerous deficiencies in the honeycomb-like hierarchical nitrogen-doped framework. The phenomenon proved that Li preferentially deposits in the deficiencies during the cycling process, and deficiencies can offer more nucleation sites, which is beneficial to the nucleation/deposition of lithium. Wu et al. proposed to introduce dual additives consisting of LiAsF_6_ and fluoroethylene carbonate into commercial carbonate electrolytes [73]. In situ Raman proved that the additive can regulate Li nucleation and growth, resulting in homogeneous and less-dendritic Li deposition. Nie et al. proved the Li^+^ platting pathways based on a scaffold (polyethyleneimine @Ag@Cu) by in situ Raman [74]. As shown in Figure 3c, the in situ Raman spectrum displayed that polyethyleneimine was closely coordinated to Ag through -NH groups, and Li^+^ enabled competition with polyethyleneimine to form massive Li deposition on the lithiophilic surface of Ag; in addition, electropositive polyethyleneimine can suppress Li^+^ from its enrichment on the protuberance to suppress Li dendrite.

#### 3.1.3. In Situ Resonance Spectroscopy

(1) In situ nuclear magnetic resonance (NMR) spectroscopy

NMR is a non-destructive technology that can reveal information about the molecular dynamics of molecules and chemical structures, and is generally used in biology, chemistry, physics, materials science, medicine, food, environmental science, and other fields. With high sensitivity to explore the local environment around the nucleus, NMR can study the local magnetic field changes around the nucleus because electrons surround the nucleus, so it can qualitatively and quantitatively analyze the information of the electronic structure of the material [32]. In situ NMR can be employed to determine the conditions under which the microstructure of lithium deposition is formed and supervise the growth of the microstructure during lithium deposition in real time.

Arai et al. investigated the influences of temperature and the operation conditions of batteries on lithium metal deposition by in situ NMR [75]. Integrating its NMR signal can evaluate the amount of deposited lithium. Hsieh et al. used in situ NMR to quantify irreversible lithium losses in batteries, differentiating losses caused by SEI formation and the “dead lithium” portion, and proving a distribution of lithium metal microstructures on electrodes [76]. In situ NMR showed that the ^7^Li NMR chemical shift and peak intensities of lithium deposition signals are lower, which demonstrates that lithium deposition is more homogeneous.

(2) In situ/operando electron paramagnetic resonance (EPR) spectroscopy

Both NMR and EPR are resonance spectroscopies. Nevertheless, EPR is a more sensitive and highly specific technology, and enables the detection of unpaired electrons or free radicals [33]. Under the application of an applied magnetic field, the analyzed material works on the principle of electron spin rather than nuclear spin. EPR is more accurate and sensitive than NMR in exploring the microstructure of Li due to the use of microwave radiation [34]. In addition, in situ electron paramagnetic resonance imaging (EPRI) has been established to investigate the microstructure of Li.

Wandt et al. presented that employing operando EPR spectroscopy as a fresh technology can semi-quantitatively detect ions of mossy or dendritic lithium [24]. Operando EPR spectroscopy exhibited that the introduction of fluoroethylene carbonate additives can reduce the formation of mossy or dendritic lithium. Li et al. proposed an in situ grown gradient SEI layer on a pre-designed micro-hole-grid Cu reservoir to promote even deposition of lithium [77]. Operando EPR exhibited a dynamic Li deposition, clearly proving the behavior of being dendrite-free.

#### 3.1.4. In Situ Microscopy

In situ microscopy is a real-time visual analysis method, which can prove the morphology of Li dendrites and see the progress of the interface in real time. The in situ microscopy technologies reviewed here include in situ optical microscopy (OM), in situ scanning electron microscopy (SEM), in situ transmission electron microscopy (TEM), and in situ atomic force microscopy (AFM).

(1) In situ OM

OM can be used to extract detailed surface information by using transmitted light to magnify and image tiny objects that cannot be resolved by the human eye [35]. In situ OM can be applied to battery research and the real-time monitoring of lithium dendrite morphology and interfacial changes.

**Figure 4:**
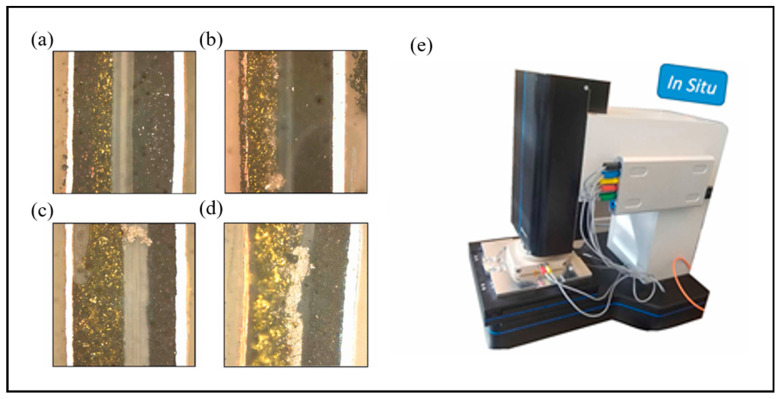
Cross-sectional in situ OM images and equipment. (**a**) Charging without Li metal deposition. (**b**) Li metal deposition without internal short circuits. (**c**) Li dendrite growth at the edge of the electrodes. (**d**) Li metal deposition due to blocking of the separator pores. (**e**) Photograph of in situ OM equipment. Reproduced with permission [61]. Copyright 2023, Elsevier.

Hogrefe et al. employed a cross-sectional in situ OM to directly observe Li metal deposition [61]. Directly observed in Figure 4 are (a) charging without Li metal deposition, (b) Li metal deposition without internal short circuits, (c) Li dendrite growth leading to internal short circuits, and (d) blockage of the separator pores causing Li metal deposition with internal short circuits. The photograph of in situ OM equipment is shown in Figure 4e. Liu et al. presented the direct observation of dendrite growth in solid-state electrolytes based on a Li_6_PS_5_Cl-polytetrafluoroethylene indicator layer [78]. In situ optical observation of the dendrite growth was carried out in a transparent battery encapsulated in a quartz mold. The time-elapsed OM images were obtained to investigate the dendrite growth inside thin solid-state electrolyte layers by in situ OM. The uniform gray layer of solid-state electrolytes was observed under an optical microscope before lithium plating. After 30 min of lithium plating, black spots appeared in the Li_6_PS_5_Cl-polytetrafluoroethylene layer, which demonstrates that dendrites have penetrated through the thin Li_6_PS_5_Cl layer. Multiple black spots appeared at the interface of the Li_6_PS_5_Cl and Li_6_PS_5_Cl-polytetrafluoroethylene layers, demonstrating that there are multiple ways of dendrite growth. The black-colored regions continued to penetrate deeper into the Li_6_PS_5_Cl-polytetrafluoroethylene layer as plating continued.

Although in situ OM technology can be employed to monitor lithium dendrite morphology, it has some limitations, such as restricted spatial resolution, that the observed sample needs to be larger than the nanometer scale, that it is limited to studying the sample surface, and opaque materials cannot be imaged.

(2) In situ SEM

In situ SEM can conduct real-time imaging through secondary electrons or backscattered electrons generated when the electron beam scans the material surface, which can visualize the morphology of the electrodes at the microscopic scale during the battery charging and discharging process [36].

**Figure 5:**
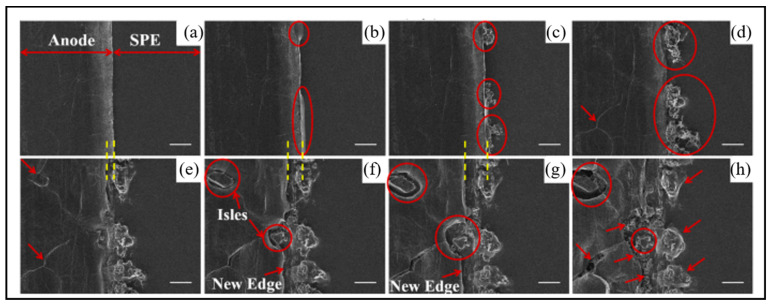
In situ SEM images obtained during cycling. (**a**) At the beginning of cycling. (**b**) After 13 h of cycling. (**c**) After 14.5 h of cycling. (**d**) After 3 days of cycling. (**e**) After 5 days of cycling. (**f**) After 7 days of cycling. (**g**) After 8 days of cycling. (**h**) After 14 days of cycling. Reproduced with permission [79]. Copyright 2019, Nature Portfolio.

Golozar et al. acquired portraits of the lithium anode surface by in situ SEM to observe lithium dendrite growth during cycling [79]. Figure 5 exhibits the in situ SEM portraits acquired as the cycle time increases. As shown in the arrows and circles, the anode surface is smooth at the beginning of the cycle. After a few hours of cycling, dendrites begin to form. After further cycling, dendrites grow further. After seven days of cycling, two isles begin to appear. Further cycling exhibits the appearance of a new edge on the anode and depletion of lithium near the isles, but it does not exhibit sustained growth of the dendrites that appeared at the beginning of the cycle. Dendrite growth on the new edge can be observed after 14 days of cycling.

Tang et al. employed in situ SEM to study the nucleation and growth of lithium dendrites [80]. In situ SEM images during Li deposition obviously indicate that whisker growth starts at the base because the morphology of the whisker tip does not change when the whisker outstretches from the base.

(3) In situ TEM

TEM is a tool for imaging and analysis that employs high-energy electron beams to excite elastic or inelastic electrons to penetrate the sample [37]. In situ TEM can dynamically monitor the microstructure at the atomic level and nanoscale in real time, and carry out the real-time monitoring of lithium dendrite morphology during cycling. 

Diaz et al. exploited a unique in situ TEM method to directly observe the lithium deposition process [60]. The in situ TEM images describe the process of lithium growth. In the beginning, the lithium dendrites grow vertically, and the vertical growth stops after 13.9 s, and horizontal growth becomes the main direction of lithium dendrite expansion. 

**Figure 6:**
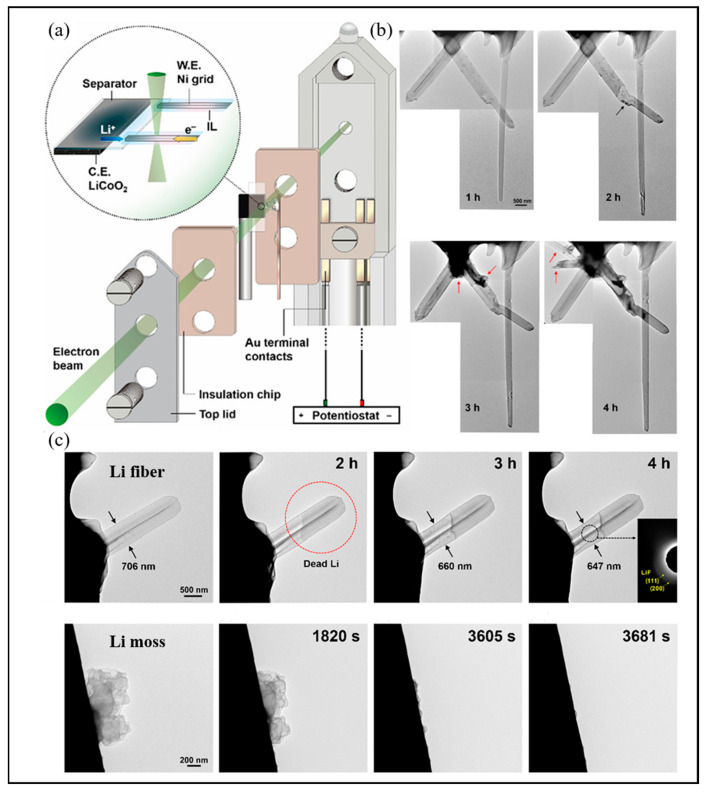
Li metal electrodeposition–dissolution monitored by in situ TEM. (**a**) Schematic diagram of the battery for in situ TEM measurements. (**b**) as indicated by the arrow: In situ TEM observation of the Li fiber growth process. (**c**) In situ TEM observations of the Li fiber and moss dissolution process. Reproduced with permission [25]. Copyright 2021, Wiley.

Chen et al. monitored the Li metal electrodeposition–dissolution by in situ TEM [25]. Figure 6a shows the in situ TEM setup used in this study. The Li fiber growth process observed by in situ TEM is shown in Figure 6b. During the electrodeposition process, the dendrite growth is primarily at the root of the deposits, and the shape of the Li tips holds constant. Figure 6c exhibits the anodic dissolution process by in situ TEM. It can be obviously observed that the dissolution of a Li fiber begins near the root segment because it becomes more electronically transparent. The circle shows that the dead Li forms due to partial stripping of the root. Furthermore, the solid electrolyte interphase shell shrinks bit by bit as time goes on, which means that it is empty in nature. Unlike fibers, in situ TEM images of mossy lithium deposits’ stripping process demonstrate that most mossy deposits disappear easily because of electrochemical dissolution. 

(4) In situ AFM 

AFM is a novel probe microscope technology exploited on the basis of scanning tunneling microscopy, which has the advantages of small intrusion, high spatial resolution, flexible application, diverse functions, and so on, and has been widely applied in surface/interface analysis [35,81]. When the probe approaches the surface of the materials, the interaction between the material and probe leads to the deformation of the cantilever [63]. Three-dimensional micro-topography images of the material surface can be obtained through exploring the deformation. In situ AFM can monitor the pathways of lithium deposition and prove the interface behavior between electrode and electrolyte at the nanoscale.

**Figure 7:**
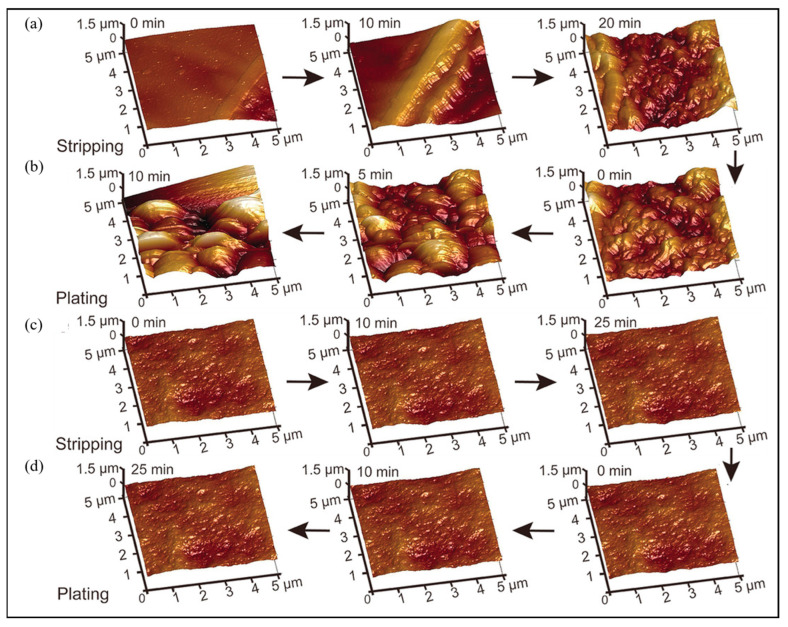
In situ AFM images. The pristine Li anode during stripping (**a**) and plating (**b**) processes. Li anode modified with the Li polyacrylic acid solid electrolyte interphase layer during stripping (**c**) and plating (**d**) processes. Reproduced with permission [82]. Copyright 2018, Wiley.

Li et al. confirmed by in situ AFM that the high elasticity of the Li polyacrylic acid solid electrolyte interphase layer can solve the dynamic lithium plating/stripping processes through adaptive interface adjustment [82]. The AFM images of the anode during the plating/stripping processes are shown in Figure 7. Many dendrite structures begin to grow on pristine Li plates over time owing to the uneven stripping of Li. In contrast, a flat surface remains during the lithium stripping/plating process on the Li anode modified with the Li polyacrylic acid solid electrolyte interphase layer. 

Lang et al. employed in situ AFM to study the morphological evolution at interfaces of Li/electrolytes under working batteries [83]. In situ AFM results demonstrate that in the electrolytes with relatively high/low concentrations of LiNO_3_, the non-uniform solid electrolyte interphase with dispersed nanoparticles is formed. 

#### 3.1.5. In Situ/Operando Neutron Technology

In situ/operando neutron technology utilizes the interaction between the neutron beam and the nucleus, has the characteristics of high sensitivity, strong penetration, and being non-destructive to lithium, and is used to reveal the evolution of crystal structure during cycling, reveal real-time electrode surface changes, and monitor the distribution and transport path of lithium [84]. The in situ/operando neutron technologies reviewed in this paper include in situ/operando neutron depth profiling (NDP), in situ/operando neutron imaging (NI), and operando small-angle neutron scattering (SANS).

(1) In situ/operando NDP

In situ/operando NDP is a noninvasive neutron analysis technology, based on the interaction of an incident neutron beam with certain elements to determine the spatial distribution of specific isotopes by neutron capture reactions, which can quantitatively monitor the evolution of the sedimentary phase of lithium metal and the kinetic behavior of lithium ions during deposition/dissolution [59].

**Figure 8:**
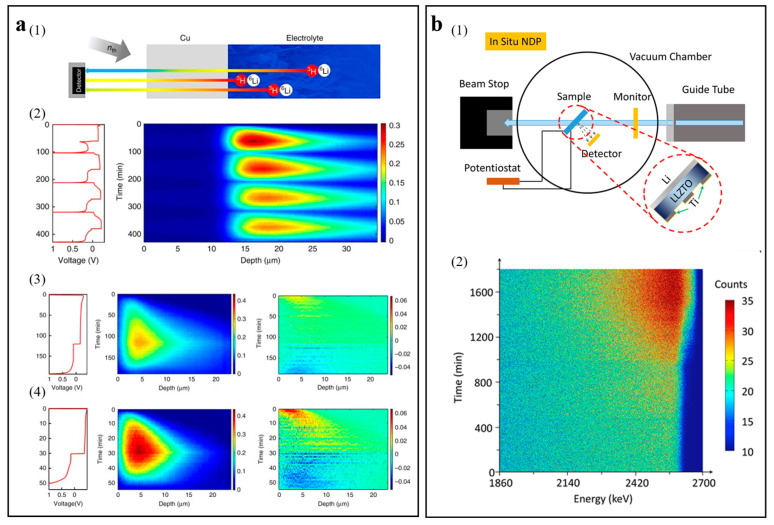
(**a**) Schematic diagram of the operando NDP device and Li density profiles of operando NDP. (1) Schematic diagram of operando NDP device. (2) Li density profiles of operando NDP during the cycle. (3) Operando NDP of the first plating and stripping cycle at 0.5 mAh cm^−2^. (4) Operando NDP of the first plating and stripping cycle at 2 mAh cm^−2^. Reproduced with permission [85]. Copyright 2018, Nature Portfolio. (**b**) Schematic of the in situ NDP equipment and the contour map of in situ NDP spectra. (1) Schematic diagram of in situ NDP device. (2) In situ NDP spectral contour map of cycling collected every 3 min. Reproduced with permission [26]. Copyright 2019, Elsevier.

Lv et al. utilized operando NDP in combination with microscopic technology, quantitatively investigating the functional relationship between lithium-ion density and depth in metal lithium [85]. A schematic diagram of the operando NDP device is shown in Figure 8(a1). Figure 8(a2) shows the Li density profiles of operando NDP measurements during the cycle, where the dense Li region corresponds to dense mossy Li deposition, while the low-density tail spreading depths into the electrolytes corresponds to dendrites. Figure 8(a3,a4) exhibit the effect of the current density on the Li density distribution by operando NDP measurements, which proves that the greater current density leads to more compact lithium deposition.

Li et al. utilized in situ NDP to study the behavior of lithium deposition in a Li|Li_6_._4_La_3_Zr_1_._4_Ta_0_._6_O_12_ (LLZTO)|Ti (three-dimensional structure electrode) solid-state battery [26]. Figure 8(b1) shows a schematic diagram of the in situ NDP device. The contour map of in situ NDP spectra is shown in Figure 8(b2). The number of counts at 2595 keV grows fast, which explains that the three-dimensional structure Ti electrode can facilitate the lithium to grow in the void space and regulate the behavior of the lithium deposition.

(2) In situ/operando NI

Neutrons can be utilized for imaging purposes, where they are selectively decayed through absorption and scattering. The neutron beam can easily penetrate the material due to the fact that the neutron has no charge. The neutron is sensitive to light elements, leading to more obvious observation in the image [40]. As a 2D/3D imaging technology, in situ/operando neutron radiography/tomography (NR/NT) can promote the monitoring of the crystal structure of the electrode changes and the mechanism of lithium deposition as well as distribution during cycling.

**Figure 9:**
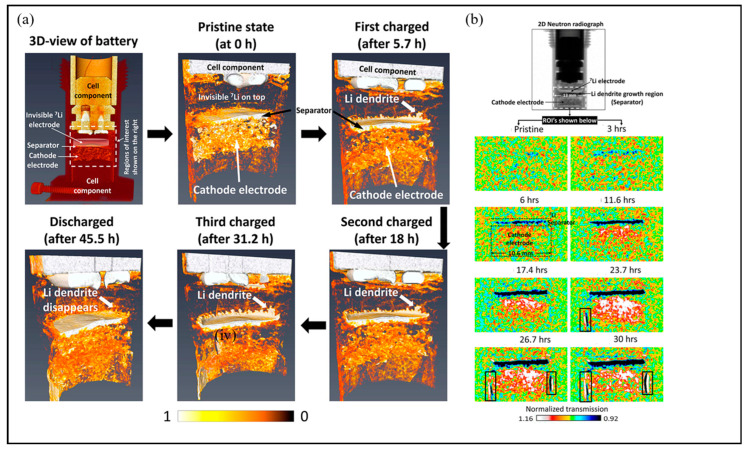
3D NT and 2D NR images. (**a**) The Li distribution 3D evolution. (**b**) The Li distribution 2D evolution. Reproduced with permission [62]. Copyright 2019, American Chemical Society.

Song et al. applied operando NR (2D) and operando NT (3D) to investigate the dynamic distribution of Li in real time [62]. Figure 9a shows the evolution of the Li distribution by 3D tomography provided after each charge and discharge step. At the original state, an almost uniform distribution of lithium can be observed. At different stages of charging, the marginal dendrites of lithium dendrites grow gradually. At the end of the discharge, this dendritic lithium disappeared, and these observations show that some of the lithium dendrites grew along the path of minimum resistance. Figure 9b shows time-resolved in situ NR, which can prove the dynamic features of the lithium distribution and dendrite growth in real time. After 3 h of battery charging, a small amount of lithium ions can be seen to deposit/migrate to the separator/lithium metal interface. As the charging time and charging voltage increase, the lithium ions continue to migrate to the separator/lithium metal interface. 

(3) Operando SANS

SANS is the scattering of neutrons in real space near the zero beam after the incident on the sample. If there is an inhomogeneous structure in the sample that is larger than the atomic distance, that is, there is a neutron scattering length density difference, then the corresponding signal contrast will appear in the detector [41]. Hence, SANS is an approach for detecting sample unevenness at the nanoscale and has no signal response to even materials. Operando SANS is quite suitable for monitoring the evolution of Li dendrites. The SANS intensity curve can precisely reflect the microstructure changes caused by the growth of Li dendrites.

**Figure 10:**
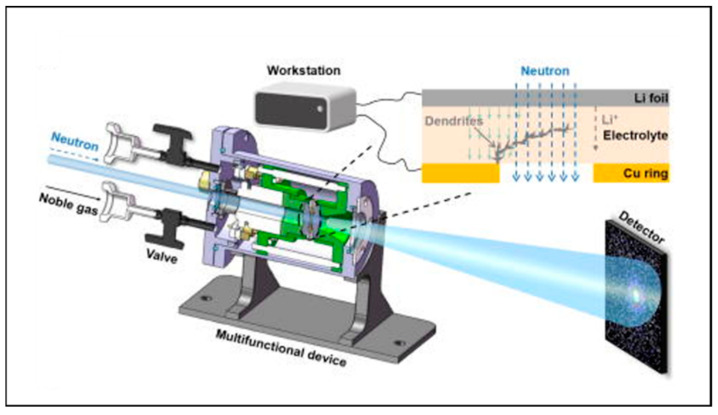
Schematic diagram of the operando SANS device. Reproduced with permission [86]. Copyright 2022, AIP Publishing.

Yang et al. employed operando SANS technology to achieve the monitoring of nanoscale lithium dendrite growth in a Li_6_._5_La_3_Zr_1_._5_Nb_0_._5_O_12_ solid electrolyte in real time [86]. The schematic diagram of the operando SANS experimental setup is shown in Figure 10. By fitting the SANS intensity curve, the roughness of the lithium dendrites can be estimated with fitting parameters. The method supplies different viewpoints to comprehend the growth mechanism of lithium dendrite. It is found that the growth of lithium dendrites is a complex dynamic evolution process of contention between self-healing and lithium dendrite growth instead of a simple accumulation process.

#### 3.1.6. Combination of Various In Situ Characterizations

In order to better study the growth mechanism of lithium dendrites, researchers consider using the above in situ characterization methods in combination. Liu et al. adopted the in situ AFM to study the morphology evolution and dynamic processes of the lithium anode at the nanoscale under additive manipulation [87]. Then, in situ OM was used to further observe the morphology of the lithium dendrite cycle. Next, in situ Raman was conducted to reveal the chemical composition formed on the lithium anode. Lu et al. proposed Ag nanoparticles encapsulated inside nitrogen-doped carbon hollow spheres with heterogeneous lithiophilicity to promote even lithium deposition [68]. The morphology evolution of the lithium anode can be studied by in situ OM. Then, in situ XRD was used to investigate lithium nucleation and growth. Li et al. presented a fresh concentration-gradient SEI layer to induce even lithium deposition [77]. In situ XRD was used to study the main lithium stripping and plating process. Next, operando EPR was carried out to further study the lithium nucleation and kinetics process. Li et al. observed the lithium dendrites growth in a Li|LLZTO|Ti (three-dimensional structure electrode) solid-state battery by self-designed in situ SEM [26]. The morphology difference can be clearly seen from the in situ SEM characterization, but the interface cannot be quantitatively analyzed. Then, in situ NDP was utilized to study the behavior of lithium deposition.

#### 3.1.7. Industrial Application of In Situ Characterizations

Further evolution of the in situ characterization of lithium dendrites should pay attention to applying these techniques in industrial battery manufacturing and testing scenarios. Gotoh et al. investigated the in situ NMR characterization of the pouch battery [88]. The deposition of the lithium dendrite is related to the diffusion rate of lithium in each component of the battery. In situ NMR characterization was used to assess the transfer rate of lithium to carbon. Therefore, in situ NMR of the pouch battery can be used to analyze the phenomenon of lithium deposition on the anode. Zhou et al. demonstrated the lithiation process on anodes in the pouch battery by using in situ NI [89]. The variation in in situ NI contrast can be correlated to the lithiation state in the actual pouch battery structure. The development of in situ characterization plays a crucial role in the testing and study of the failure mechanism of practical industrial batteries. In addition, unlike in situ characterization of batteries at a laboratory level, industrial level batteries require sampling testing and data statistical analysis, which increases the difficulty and cost of in situ characterization testing. Therefore, it is necessary to further develop the popularity of in situ characterization technology and reduce costs.

### 3.2. Sensors

As a result of the urgent need for a more convenient method to monitor lithium dendrites, more and more researchers have begun to pay attention to battery sensors. Battery sensors have the advantages of sample intactness, easy operation, detection portability, real-time response, and intelligence, making them monitor lithium dendrites in real time. Most of all, the sensor can be implanted in a battery with non-destructiveness, enabling extremely precise tests [42]. The battery sensors reviewed in this paper include optical fiber sensors, gas sensors, and membrane sensors.

#### 3.2.1. Optical Fiber Sensor

The optical fiber sensor has the merits of anti-electromagnetic interference, small dimension, low weight, large bandwidth, great sensibility, and so on, which can be widely employed to detect strain, temperature, and pressure [90]. Optical fiber sensors have already been used to monitor parameters inside batteries presently.

**Figure 11:**
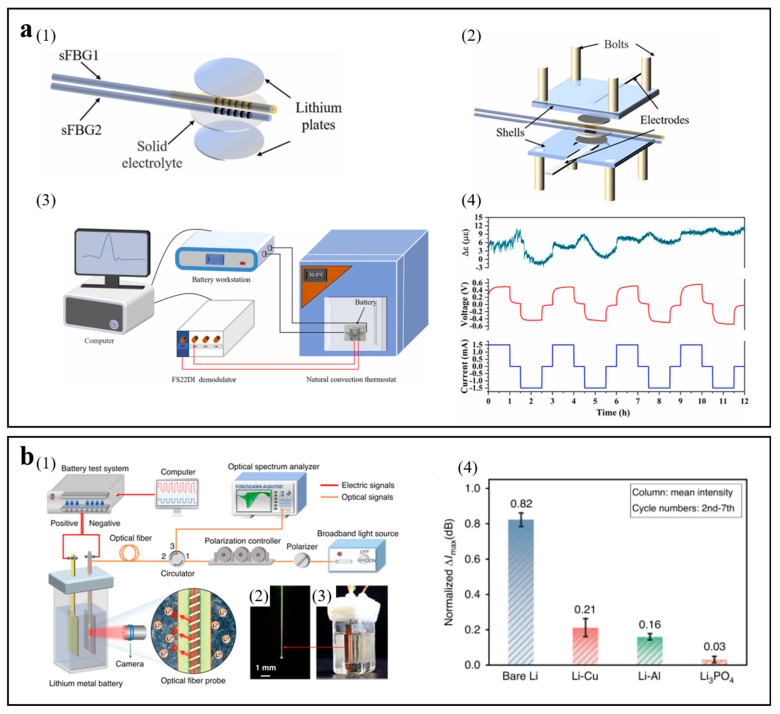
(**a**) Experimental setup diagram and external strain variations monitored by short fiber Bragg gratings. (1) Illustration diagram of the location of the optical fiber sensors. (2) Illustration diagram of the battery. (3) Illustration diagram of the battery monitoring the temperature and strain. (4) External strain variations monitored. Reproduced with permission [27]. Copyright 2022, Elsevier Science SA. (**b**) Illustration diagram of the sensing monitoring system and analysis of the lithium plating. (1) Schematic diagram of the evanescent optical fiber sensing system. (2) Photo of the optical fiber sensing probe. (3) Photo of the lithium symmetrical battery. (4) Comparison of the normalized Δ*I*_max_ values. Reproduced with permission [91]. Copyright 2024, Springer Nature.

Xi et al. presented the implantation of short fiber Bragg gratings into the battery to monitor internal strain and temperature information in real time [27]. Lithium dendrites can be resoundingly monitored during cycling processes. This method can monitor the working state of solid-state batteries and has important significance and value for battery safety detection in a variety of domains. Figure 11(a1) and Figure 11(a2) show the schematic diagram of the location of the optical fiber sensor and the schematic diagram of the battery. The schematic diagram of the experimental device for monitoring the temperature and strain of the battery in real time is shown in Figure 11(a3). The strain changes recorded by short fiber Bragg grating in the battery are exhibited in Figure 11(a4). It can be seen that the variation of strain in the first two cycles is great, the variation of strain in the last three cycles is little, and the maximum variation of strain during the entire process is 11.5 με. The main cause of variation of strain may be the dendrites’ formation on the surface of the lithium during the process of cycling. The internal strain of the battery does not change much in the late cycle, which demonstrates that the dendrites have gradually developed a constant shape.

Han et al. proposed the implantation of a tilted fiber Bragg grating sensor into an operational lithium metal battery that is inserted close to the electrode surface without interfering in its operation [91]. Monitoring lithium dendrite growth has been accomplished due to the optical resonances of the tilted fiber Bragg grating sensor. This novel method can provide extensive direction for the design of a better monitoring battery. The schematic diagram of the experimental device of the electrochemical optical fiber sensing system for monitoring the ionic concentration at the interface between the electrolyte and electrode is shown in Figure 11(b1–b3). The adjustment behavior of lithium plating can be reflected by a tilted fiber Bragg grating sensor monitoring the mass transport kinetics at the interface of the electrode. Figure 11(b4) shows the normalized Δ*I*_max_ values, and then the normalized results are analyzed. The normalized Δ*I*_max_ values for Li-Cu and Li-Al anodes are lower than the bare lithium anode. For the Li-Li_3_PO_4_ anode, the normalized Δ*I*_max_ value decreased observably. Obviously, the surface of the bare lithium anode shows the highest concentration gradient, intensifying the formation of lithium dendrites in the bulk electrolyte.

#### 3.2.2. Gas Sensor

Safety issues of lithium batteries at an early stage can be detected by the special gas exploration of lithium batteries. Jin et al. presented for the first time a method for detecting micron-scale lithium dendrites using H_2_ gas capture [43]. The spontaneous reaction between common electrode polymer binders and lithium metal can generate H_2_ gas at an early stage. H_2_ gas sensors can capture H_2_ gas as a valid indicator of lithium metal anomalies. The method can work without changing the commercial lithium battery structure. Yan et al. used a single dual-mode (direct and alternating current modes) SnO_2_ sensor to detect H_2_ gas, achieving the accuracy of the lithium battery safety warning system at an early stage [92]. 

#### 3.2.3. Membrane Sensor

The deformability of membrane sensors makes them suitable for curved and irregularly shaped batteries, which can guarantee great contact between the battery and the sensor and afford precise distribution of stress. The membrane sensor can cover the wide region within the battery, afford abundant spatial distribution information, and monitor the distribution of stress in real time. 

**Figure 12:**
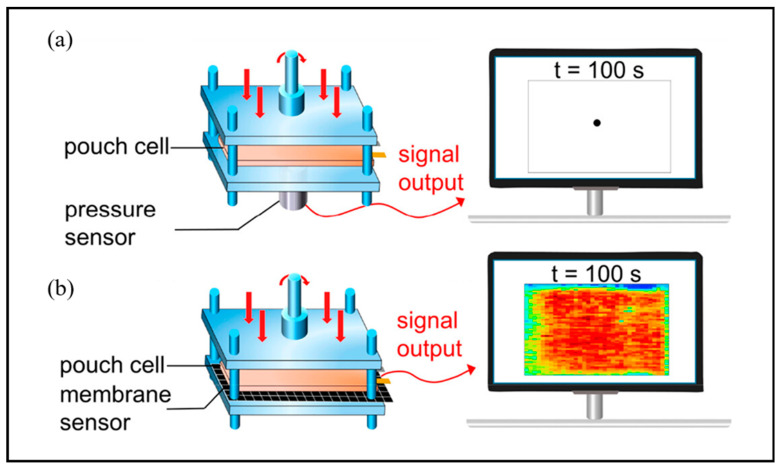
Comparison of stress sensors. (**a**) Single-point stress sensor and data portrait. (**b**) Membrane stress sensor and data portrait. Reproduced with permission [44]. Copyright 2023, American Chemical Society.

Li et al. investigated the value of membrane sensors in battery stress monitoring and battery performance under uneven stress, as well as the effects of local high pressure and the consequent pressure gradient on lithium dendrite growth [44]. Figure 12 shows pressure measurement using a membrane sensor, which is placed below the battery. Due to the merits of being flexible and lightweight, membrane sensors can suit complicated dynamic environments and respond quickly to variations in local pressure. The study shows that the local high pressure influences the overall stress evolution in the battery and emphasizes the problems prompted by stress gradients, which are not beneficial to the stability of electrode structures and facilitate the growth of lithium dendrites.

## 4. Conclusions and Perspective

In summary, revealing the dendrite growth mechanism is important for the design of safe and high-performance LIBs. Uneven charge distribution during battery operation leads to uneven deposition of lithium ions, forming lithium dendrites on the anode surface, resulting in battery capacity degradation, short circuit, and even thermal runaway. Therefore, in-depth analysis of the growth mechanism of lithium dendrites has important guiding significance for the design of high-safety and high-performance lithium-ion batteries. However, ex situ characterization is difficult to monitor the lithium dendrite growth in real time. Therefore, it is urgent to develop a detection technology that can monitor the growth state of lithium dendrites in real time during the actual working process of the battery. In this review, we summarize the real-time monitoring techniques for lithium dendrite growth from the literature, including in situ/operando characterization technologies and sensors. The significance of the research lies in the following three points. Firstly, real-time monitoring of the interface or internal state of the battery avoids the safety hazards of the battery. Secondly, a more intuitive observation of the electrochemical reactions inside the battery has proposed new solutions for deeper material preparation or process development. Thirdly, through real-time monitoring technologies, the subsequent usage of the battery can be predicted, such as charging status, capacity, life, and so on. In addition, real-time monitoring technologies still have certain limitations, such as testing duration and cost issues. However, in the face of the significant importance of studying the internal reaction mechanisms of high-safety and high-performance batteries, real-time monitoring technologies are essential and urgently need to be vigorously developed. We hope that this review will provide introductory guidance for the application of various nano-engineering techniques and operational characterization techniques to understand the dynamic evolution of dendrites in metal battery systems. We hope that this review can provide an introductory guide for the study of lithium dendrites.

## Figures and Tables

**Table 1:** A summary of real-time monitoring technologies.

Technologies	The Main Uses	Merits	Drawback	Ref.
In situ XRD	Phase transition and structural change	No destruction, high detection speed, high precision, and no contamination	Not applicable to amorphous materials and not suitable for direct observation morphology	[28]
In situ XPS	Elemental composition	Fast speed, no damage, high precision, and analysis depth is a few nanometers	Not applicable to the detection of overall composition	[29]
In situ Raman	Crystallinity, chemical structure, phase, and molecular interactions	Non-destructive, applicable to amorphous or weakened crystallized compounds	Not applicable to Li metal	[30,31]
In situ NMR	The information of electronic structure is analyzed qualitatively and quantitatively	Non-destructive, high-sensitivity	Expensive and long experiment time	[32]
In situ EPR	Detection of unpaired electrons or free radicals	Non-destructive, higher sensitivity, and accurate	Complex operation	[33,34]
In situ OM	Morphology evolution	Simple operation, and low cost	Low spatial resolution, the sample size is larger than nanometers, and only studies the sample surface	[35]
In situ SEM	Micromorphology	Large depth of field and high-definition	Harsh operating conditions	[36]
In situ TEM	Micromorphology	Extra-high resolution	Sample thickness is limited and harsh operating conditions	[37]
In situ AFM	Surface morphology and structure information and surface roughness information	Small intrusion, high spatial resolution, flexible application	Slow scanning speed	[38]
In situ NDP	Real-time distribution and migration of lithium ions	High sensitivity, strong penetration, and non-destructive to lithium	Under vacuum or pressure atmosphere	[39]
In situ NI	Lithium dynamic distribution	Sensitivity to light elements	Expensive	[40]
Operando SANS	Structure at the nanoscale	Sensitivity to light elements, identification of isotopes, and strong scattering of magnetic moments	Low neutron source brightness	[41]
Optical fiber sensor	Detection of strain, temperature, and pressure	Anti-electromagnetic interference, small dimension, low weight, large bandwidth, great sensibility	Expensive and high environmental requirements	[42]
Gas sensor	Detection of generated gas	High sensitivity, quick response	Expensive, poor anti-interference	[43]
Membrane sensor	Detection of stress distribution	Deformability, simple structure, fast response speed, long service life	Precision limitation, poor anti-interference	[44]

**Table 2:** Comparison of in situ characterization techniques and their corresponding ex situ characterization techniques.

Technologies	Ex Situ	In Situ
XRD	Tests are carried out at the end of the reaction or at specific stages The state of the electrode may change during disassembly, washing, and other operations, affecting the accuracy of XRD peaks	Real-time monitoring of electrode structure changes during reaction or charge and discharge process
XPS	Evaluation of electrode/electrolyte interface chemical structure at the end of the reaction	Evaluation of chemical changes at the interface under electrochemical conditions
Raman	Only the end product can be tested	Monitoring the intermediate products, the reaction process
NMR	Investigating the reactants and products of the reaction	Different components of the same battery can be studied in different charging states Instantaneous states and the dynamic processes occurring in real time can be investigated
EPR	Free radicals are detected by trapping them in probe molecules The sample needs to be tested immediately	Real-time monitoring of electron spin signals during electrochemical reactions
OM	Morphology of electrode before and after reaction	Monitoring the growth morphology of lithium dendrites in real time
SEM	Pretreatments such as sample fixation and slicing may result in changes in the original state of the sample	Real-time observation in close proximity to the actual environment of the sample Capturing the process of sample change under specific conditions
TEM	Microstructure before and after reaction	Monitoring the microstructure evolution during the chemical reaction
AFM	Morphology and mechanical properties before and after reaction	The evolution of electrode morphology and mechanical properties can be dynamically observed
NDP	Analyzing the composition of electrode material	Analyzing the distribution and migration of lithium ions in real time
NI	The neutron beam penetrates material for imaging	Monitoring of lithium deposition and distribution during the cycle
SANS	Characterization of nanoscale material unevenness	Investigating the dynamic behaviors and the evolution of Li dendrites in real time
